# The Polyherbal Functional Ingredient Containing Ginger, Chinese Date, and Wood Ear Mushroom Protects against Dementia following Metabolic Syndrome

**DOI:** 10.1155/2023/9911397

**Published:** 2023-08-02

**Authors:** Thuntiva Nakyam, Jintanaporn Wattanathorn, Wipawee Thukham-mee, Supaporn Muchimapura

**Affiliations:** ^1^Department of Physiology and Graduate School (Neuroscience Program), Faculty of Medicine, Khon Kaen University, Khon Kaen, Thailand 40002; ^2^Integrative Complementary Alternative Medicine Research and Development Center in Research Institute for Human High Performance and Health Promotion, Khon Kaen University, Khon Kaen, Thailand 40002; ^3^Department of Physiology, Faculty of Medicine, Khon Kaen University, Khon Kaen, Thailand 40002

## Abstract

The anti-dementia effect following ischemic stroke with metabolic syndrome (MetS) of the polyherbal functional ingredient comprising ginger, Chinese date, and wood ear mushroom (GCJ) was hypothesized due to its neuroprotective effect against stroke. This study was performed to test this hypothesis and to explore the underlying mechanism. Male Wistar rats weighing 180-220 g were induced metabolic syndrome (MetS) with a 16-week high-carbohydrate high-fat diet (HCHF) feeding. The rats with MetS characteristics were orally administered GCJ at various doses (GCJ100, GCJ200, and GCJ300 mg kg^−1^ BW) 21 days pre-induction and 21 days post-induction of reperfusion injury (I/R) at the right middle cerebral artery (MCAO). Memory was evaluated every 7 days during the study period. At the end of the study, neuron density, AChE activity, and the expressions of eNOS, BDNF, and pERK/ERK in the prefrontal cortex, and hippocampus were also determined. MetS rats with GCJ treatment improved memory impairment, enhanced neuron density, and increased the expressions of eNOS, BDNF, and pERK/ERK but suppressed AChE in both areas. Therefore, the anti-dementia effect following ischemic stroke with metabolic syndrome of GCJ may involve the improvement of AChE, eNOS, BDNF, pERK/ERK, and neural plasticity. However, this required confirmation by clinical study.

## 1. Introduction

Cognitive impairment is one of the most common problems in stroke survivors. Around 77% of stroke survivors have problems with memory [1], and this condition is aggravated by metabolic syndrome. It has been revealed that cognitive impairment is associated with neurodegeneration induced by oxidative stress, poor glucose, and poor blood supply [2–5]. In the comorbidity of cerebral ischemia stimulated by ischemic stroke and metabolic syndrome, oxidative stress is higher than in ischemic stroke deprived of metabolic syndrome. This elevation is beyond the endogenous buffering capacity [2, 3]. In addition to oxidative stress, inflammation is also regarded as a key driver of secondary neurodegeneration and memory impairment following stroke [6]. Due to the vital role of oxidative stress and inflammation in the pathophysiology of memory impairment, the beneficial effects of the substances possessing antioxidant and anti-inflammation activities have gained much attention [2–4].

The polyherbal recipe containing ginger (*Zingiber officinale* Roscoe), Chinese date (*Ziziphus jujuba* Mill), and wood ear mushroom or Jew's ear mushroom (*Auricularia auricula-judae*) has been long-term used in Thailand, especially in the Thai-Chinese descents, it has been long term widely used in the Thai-Chinese descents for clearing and caring for blood vessels. Accumulative lines of evidence also reveal that the herbs just mentioned also possess the biological assessment related with the pathophysiology of stroke, metabolic syndrome, and memory impairment such as antioxidant and anti-inflammation [7–15]. In addition, our recent study also clearly demonstrates that GCJ exhibits a cerebroprotective effect against ischemia-stroke with metabolic syndrome (MetS) [16]. Owing to the potential benefits of the aforementioned herbs on vascular, the buffering capacity on oxidative stress and inflammation together with the cerebroprotective effect against ischemic stroke, the positive variation effect of GCJ on memory impairment. To the best of our information, there is no scientific evidence to support this. Therefore, we aimed to determine whether this formulation improved memory performance and protected against neurodegeneration in cerebral ischemic rats with metabolic syndrome. Moreover, the possible mechanism behind the effects of GCJ was also examined.

## 2. Materials and Methods

### 2.1. Preparation of the Polyherbal Functional Ingredient Containing the Mixture of GCJ

All herbs used in this study were collected between October 2020 and January 2021. After the authentication process, the polyherbal functional ingredient was prepared by mixing an aqueous extract of ginger, Chinese date, and wood ear mushroom (ratio 1 : 1 : 1). Then, the chromatogram analysis of the functional ingredient was performed by using high-performance liquid chromatography (HPLC; Waters Co., Milford, MA), equipped with pump control module II Waters® 515, a sample of 20 *μ*L loop with Rheodyne injector, and an array detector of photodiode Waters® 2998. Compounds were separated via a Poroshell® 120 EC-C18 column (250 × 4.6 mm id, 4.0 *μ*m; Agilent Technologies, USA), joined with a Poroshell® 120 EC-C18 (5 × 4.6 mm id, 2.7 *μ*m, guard column; Agilent Technologies, USA), and maintained at room temperature. The mobile phase consisting of 70 percent acetonitrile (solvent A) and 0.1 percent formic acid (solvent B) in deionized (DI) water was applied with a gradient, as described in the following: 70% A (0-17 min), 100% A (18-20 min), and 10% A (20.5-25 min). A tested sample was filtered through a 0.22 *μ*m millipore membrane, and 20 *μ*L of the filtered sample was injected with a flow rate of 2 mL/min. The chromatographic profile was detected by using a UV detector at 280 nm and 370 nm. The data was analyzed using EmpowerTM3.

### 2.2. Experimental Protocol

All experimental procedures used in this study were approved by the Animal Ethics Committee of Khon Kaen University (Record number IACUC-KKU 42/63). A male Wistar rat with basal weight ranging from 180 to 220 was sourced from the Northeast Laboratory Animal Center, Khon Kaen University, Thailand, and was used as an experimental animal. All rats were housed in a laboratory with optimal settings of 12 hours of the light-dark cycle, water and food at ad libitum, and a temperature maintained at 23 ± 2°C. Then, rats were randomly selected and placed into either of the 8 experimental groups (6 rats per group). The description of the MetS-induced HCHF diet used, and the inclusion criteria for the rats that developed MetS characteristics were in accordance with our recent study. The details of the animal grouping are described in Table 1.

All administrations of the assigned substances were performed once daily, 21 days before and 21 days after MCAO. Spatial memory was carried out using the Morris water maze test whereas, nonspatial memory was performed using the novel object recognition test. All memory assessments were carried out every 7 days throughout the experimental period. At the end of the study, AChE activity and the expressions of brain-derived neurotrophic factor (BDNF), endothelial nitric oxide synthase (eNOS), and signal transduction molecules of memory such as a total extracellular regulated kinase (ERK) and a phosphorylated form of extracellular regulated kinase (pERK) in prefrontal cortex and hippocampus were determined.

### 2.3. Focal Ischemic-Reperfusion Injury Induction

For the induction of cerebral ischemia reperfusion injury, the surgical occlusion of the right middle cerebral artery (MCAO) was temporarily carried out. Rats were administered anesthesia using pentobarbital sodium (60 mg kg^−1^ BW). Each rat was exposed to a 90-minute-occlusion of the right middle cerebral artery with a coated silicone nylon (no.4, dimension 1.5, Taiwan). At the end of an occlusion period, a nylon was withdrawn to allow the reperfusion injury [16, 17]. Similar processes were also carried out for the sham operation group, except that no filament insertion was performed.

### 2.4. Memory Assessment

#### 2.4.1. Spatial Memory Assessment

In the summarize procedure, a circular pool (147 cm in diameter) comprising 4 quadrants occupied with water at a temperature of 25 ± 1°C and 60 cm depth. To ensure enhanced the visibility in the water pool, a nontoxic milk powder was applied to the surface of the water. All experimental animals were trained to acclimatize and to remember the location of the escape platform immersed in one fixed quadrant by using extramaze cues. The latency time to find a platform was recorded and regarded as escape latency. The rats were reexposed to the similar state mentioned earlier except that an immersed platform was withdrawn, and the time spent in the quadrant previously located an immersed platform was considered retention memory [5].

#### 2.4.2. Nonspatial Memory Assessment

This test was carried out using the description of a previous experiment [18–20]. The rat to be tested was first introduced into an enclosed arena (50 cm high, 80 cm long, and 60 cm wide) under a brightly lit area with two similar objects for a 3 minutes regarded as T1 at the middle area, and it was returned to its cage after each test session. Then, it was reexposed to a 3-minute-test at 30 minutes and 6 hours after the administration of the assigned substance, but one of the objects was replaced by an unfamiliar or novel object. The objects and the device were cleaned with 70% ethanol between each trial to avoid an influence of odor. The time spent for an exploration of each object was documented and evaluated as a novel object ratio (NOR) as the following equation:

(1) $$\begin{array}{c} \text{NOR}=\frac{T_{\text{novel}}-T_{\text{familiar}}}{T_{\text{novel}}+T_{\text{familiar}}}. \end{array}$$

(*T*_novel_ represents the period of time expanded to locate for the novel object, and *T*_familiar_ represents the period of time expanded to locate for the familiar object.)

### 2.5. Histological Assessment

The rat brains were collected and subsequently fixated with a prepared solution of 4% paraformaldehyde at a pH 7.4 and a temperature of 4° Celsius. The brain tissue was then ensured to be protected in its integrity by introducing it into the 30% formalin-sucrose solution for 2-3 days. Then, a cryostat sectioning was used to prepare the brain section at 10 *μ*m thickness. All sections were taken on slides covered with 0.3% gelatine buffer and 0.05% aluminium potassium sulfate. For the staining process, slides were first air dried and rehydrated in various grades of ethanol and xylene, respectively. Crystal violet staining was then carried out by incubating the sections for 8 minutes, followed by decolorization in acetic acid, dehydration, and cover slip with Permount. The density of survival neurons in the prefrontal cortex and hippocampus was evaluated microscopically using a light microscope. The neuronal density of the viable neurons was analyzed accordingly [21].

### 2.6. Determination of Acetylcholinesterase (AChE) Activity

AChE activity was monitored using the modified spectrophotometric method of Ellman [6, 22]. Brain homogenate at a volume of 20 *μ*L was combined chemically with the reaction mixture consisting of 200 *μ*L of 0.1 mM sodium phosphate and an assembled reagent of 10 *μ*L of 0.2 M 5,5′-dithio-bis-2-nitrobenzoic acid (DNTB) and incubated at room temperature for 5 minutes. Subsequent to the elapse of the incubation time, an aliquot of 15 mM acetylcholine trichloride at a volume of 10 *μ*L was introduced and incubated for 3 minutes. Following this step, the wavelength of 412 nm was posed and the absorbance was absorbance at 412 nm was measured by applying a microplate reader [23]. The activity of AChE was computed by applying the equation represented below.

(2) $$\begin{array}{c} \text{AChE activity}=\left(\frac{\Delta A}{1.36\times 10^{4}}\right)\times \left(\frac{1}{120/230}\right)C. \end{array}$$

Δ*A* is the change in absorbance per minute; *C* is the concentration of the protein in the homogenate of the brain. Data were expressed as nmol/min mg protein.

### 2.7. Western Blot Analysis

Western blot was employed to assess the expressions of phosphorylated extracellular signal-regulated kinase/extracellular signal-regulated kinase (pERK/ERK), endothelial nitric oxide synthase (eNOS), and brain-derived neurotrophic factor (BDNF) [7, 24] in the prefrontal cortex and hippocampus. A tissue sample from the ipsilateral brain was collected and homogenized with a mixture of the extraction of 1 : 10 ratio of mammalian reagent and protease inhibitor. A tissue sample from the ipsilateral cortex was mixed and lysed in RIPA buffer comprising 20 mM Tris-HCl pH 7.5, 150 mM NaCl, 1 mM EGTA, 1 mM Na_2_EDTA, 1% sodium deoxycholate, 1% NP-40, 1 mM beta-glycerophosphate, 1 mM Na_3_VO_4_, 2.5 mM sodium pyrophosphate, 1 *μ*g/ml leupeptin, and 1 mM phenylmethanesulfonyl fluoride. The supernatant was removed and centrifuged at 4°C at 12,000 g for 10 minutes. A sample lysate of 80 *μ*g was incubated with Tris-Glycine SDS-PAGE at 95°C for 10 minutes. Then, a sample protein at a volume of 20 *μ*L was loaded on a SDS-polyacrylamide gel and alienated by sodium dodecyl sulfate-polyacrylamide gel electrophoresis. Then, the alienated proteins were transferred from gel to nitrocellulose membrane, flushed with 0.05% TBS-T, and exposed to a blocking process by incubating a membrane in a blocking buffer comprising 5% skim milk and 0.1% TBS-T for a period of 1 hour at a specific temperature of 25°C. Then, the nitrocellulose membrane was incubated with the following primary antibodies including anti-BDNF antibody (dilution 1 : 1000), anti-phospho-Erk1/2 (Thr202/Tyr204) (dilution 1 : 1000), anti-Erk1/2 (dilution 1 : 1000), anti-eNOS (dilution 1 : 1000) antibodies at room temperature for 2 hours. However, incubation with anti-*β*-actin (dilution 1 : 1000) was performed for 2 hours at a specific temperature of 25°C. At the end of the incubation, all membranes were washed with 0.05% T-PBS for 30 minutes. Then, they were mixed with secondary antibody (anti-rabbit IgG and HRP-linked antibodies at a dilution of 1 : 2000) to intensify the signal transduction. The protein bands' density was evaluated using the ECL system and the luminescent image analyzer (LAS-4000, GE Healthcare). Band densities were quantified into numerical values by using the ImageJ software system. The numeric variables obtained from the band densities of the control normal group were represented as the bands' relative density [8].

### 2.8. Statistical Analysis

All the numerical data from this study were expressed using descriptive statistics of the mean ± standard error of mean (SEM). The SPSS software version 25.0 was used for statistical analysis to determine the difference between the groups. The difference was evaluated using a one-way analysis of variance, followed by a Tukey post hoc test. The *p* regarded to assess the significant between the groups was set at less than 0.05.

## 3. Results

### 3.1. Fingerprint Chromatogram Analysis

**Figure 1:**
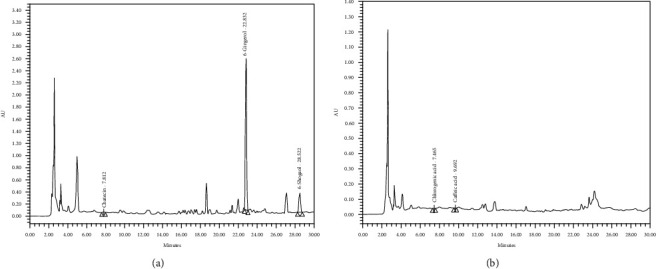
Chromatogram profiles of the functional ingredient “GCJ.” (a) Chromatogram at the wavelength of 280 nm. (b) Chromatogram at the wavelength of 370 nm.

The polyherbal functional ingredient, “GCJ”, contained 6-gingerol, 6-shogaol, catechin, chlorogenic acid, and caffeic acid at concentrations of 36.981 ± 0.62, 8.558 ± 0.19, 0.137 ± 0.04, 0.042 ± 0.007, and 0.029 ± 0.001 *μ*g/50 mg extract, respectively, as shown in Figure 1 and Table 2.

### 3.2. Memory Performance

**Figure 2:**
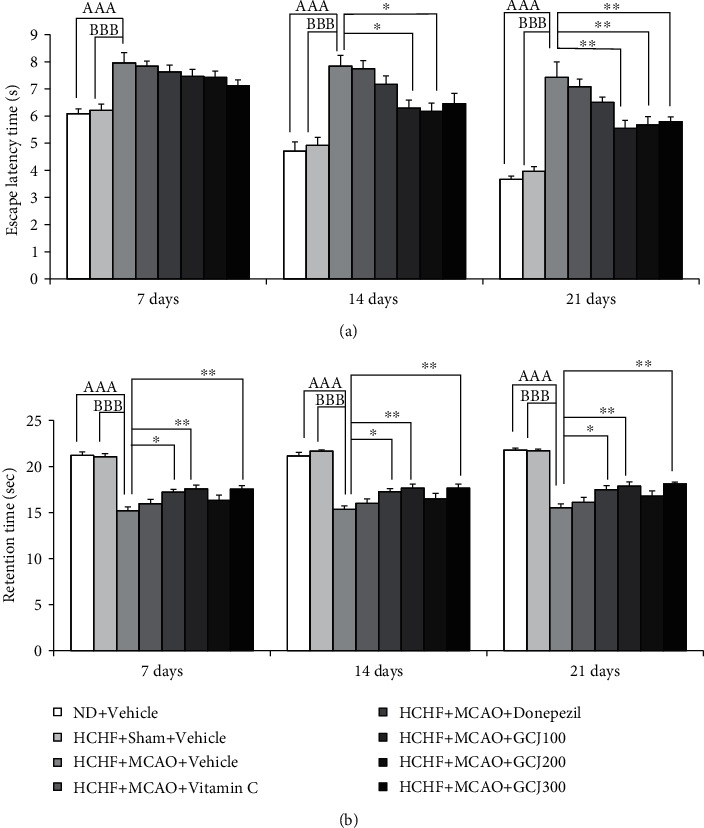
(a) The outcome produced by the herbal formulation comprising GCJ on an escape latency time. (b) The outcome produced by the herbal formulation comprising GCJ on a retention time. Data were stated as the mean ± SEM. ^AAA^ stands for statistical significance from normal diet plus vehicle at *p* < 0.001; ^BBB^ stands for *p* < 0.001, statistical significance from HCHF diet plus sham plus vehicle; ^∗^^,^^∗∗^ stand for *p* < 0.05 and 0.01, respectively, statistically significance from HCHF diet plus MCAO plus vehicle (*n* = 6 per each group).

The current data showed that HCHF-treated rats or MetS rats subjected to a sham group with vehicle showed no significant changes in both escape latency time and retention time throughout the study period, as shown in Figures 2(a) and 2(b). In this study, the memory performance was assessed after each 7 days during the period of 21 days study.  Figure 2(a) shows the effect of GCJ on the spatial memory assessed by the Morris water maze test. The sham operation and vehicle ceased to show a significant change in escape latency in HCHF-treated rats throughout the study period. MCAO significant elevation in escape latency in HCHF diet plus vehicle at 7, 14, and 21 days after MCAO (*p* < 0.001, compared with normal diet plus vehicle, and *p* < 0.001, compared with HCHF diet plus sham plus vehicle). An absence of significant change in this parameter was observed in the HCHF diet plus MCAO plus vitamin C or donepezil at 7, 14, and 21 days of treatment. GCJ at the doses of 100 and 200 mg kg^−1^ BW mitigated an increment in measured escape latency at 14 days post-MCAO (*p* < 0.05 all, compared with HCHF diet plus MCAO plus vehicle). At 21 days of treatment, an elevation of escape latency instigated by MCAO was diminished by GCJ at various doses (GCJ100, GCJ200, and GCJ300 mg kg^−1^ BW) (*p* < 0.01 all, compared with HCHF diet plus MCAO plus vehicle). The effect of GCJ on retention memory is shown in Figure 2(b). The retention time result was reduced by MCAO rats fed with HCHF and vehicle throughout the study period (*p* < 0.001 all, compared with normal diet plus vehicle; *p* < 0.001 all, compared with HCHF diet plus sham plus vehicle). Donepezil and GCJ at doses of 100 and 300 mg kg^−1^ BW significantly increased retention time in the HCHF diet plus MCAO at 7, 14, and 21 days, respectively (*p* < 0.05, 0.01, and 0.01 all, compared with HCHF diet plus MCAO plus vehicle).

**Figure 3:**
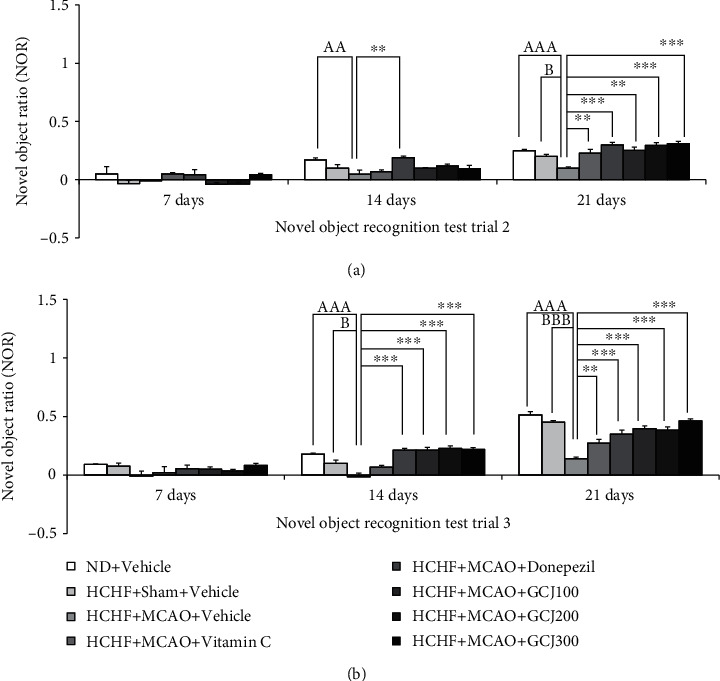
The outcome produced by the herbal formulation comprising GCJ on novel object recognition test. (a) Novel object ratio assessed at 30 minutes after the substance administration. (b) Novel object ratio assessed at 6 hours after the substance administration. Data were stated as the mean ± SEM. ^AA,AAA^ stand for *p* < 0.01 and 0.001, compared with normal diet plus vehicle; ^B,BBB^ stand for *p* < 0.05 and 0.001, compared with HCHF diet plus sham plus vehicle; ^∗∗^^,^^∗∗∗^ stand for *p* < 0.01 and 0.001, compared with HCHF diet plus MCAO plus vehicle (*n* = 6 per each group).

Figure 3(a) reveals that at 7 days after MCAO, no meaningful change in the novel object ratio (NOR) was observed in any groups when an assessment was performed 30 minutes after the administration of the assigned substance. MCAO significantly decreased the NOR of HCHF plus vehicle-treated rats at 14 (*p* < 0.01, compared with normal diet plus vehicle), and 21 days post-MCAO (*p* < 0.001, compared with normal diet plus vehicle; and *p* < 0.05; compared with HCHF plus sham plus vehicle). Only donepezil, a standard drug used for treating memory impairment, produced significant NOR in HCHF diet plus MCAO (*p* < 0.01; compared with HCHF diet plus MCAO plus vehicle). The current data demonstrated that vitamin C, donepezil, and all doses of GCJ significantly increased NOR in HCHF diet plus MCAO (*p* < 0.01, 0.001, 0.01, 0.001, and 0.001, respectively; compared with HCHF diet plus MCAO plus vehicle). The assessment of the NOR was also performed 6 hours after the administration of the tested substances, and results are shown in Figure 3(b). It was shown that no significant difference in NOR was noted in any group after 7 days of treatment. HCHF diet plus MCAO plus vehicle significantly decreased NOR at 14 and 21 days of treatment (*p* < 0.001 all, compared to normal diet plus vehicle; *p* < 0.01 and 0.001, respectively, compared with HCHF diet plus sham plus vehicle). When compared with HCHF diet plus MCAO plus vehicle, the present data revealed that donepezil and all doses of GCJ produced significant increases in NOR in HCHF diet plus MCAO (*p* < 0.001 all). However, vitamin C could significantly enhance NOR in HCHF diet plus MCAO rats only 21 days after treatment (*p* < 0.01; compared with HCHF diet plus MCAO plus vehicle).

### 3.3. Neuroprotective Effect

**Figure 4:**
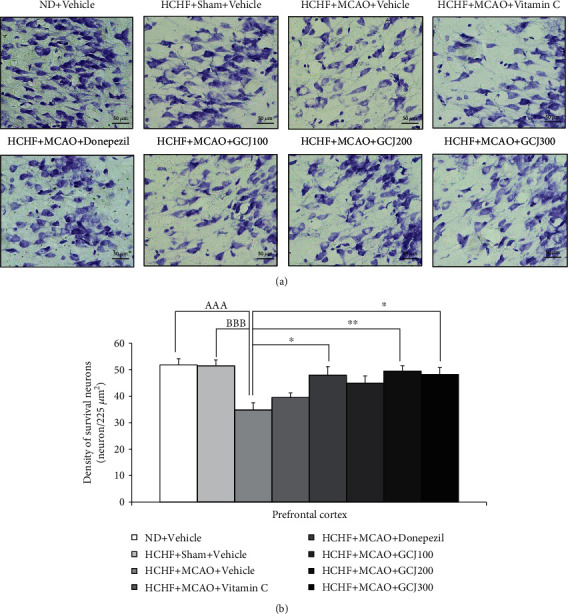
The outcome produced by the herbal formulation comprising GCJ on the survival neuron density in the prefrontal cortex. (a) Representative pictures of the prefrontal cortex stained with cresyl violet. (b) The survival of neuronal density in the prefrontal cortex. Data were as stated as the mean ± SEM. ^AAA^ stands for *p* < 0.001, compared with normal diet plus vehicle; ^BBB^ stands for *p* < 0.001, compared with HCHF diet plus sham plus vehicle; ^∗^^,^^∗∗^stand for *p* < 0.05 and 0.01, compared with HCHF diet plus MCAO plus vehicle (*n* = 6 per each group).

**Figure 5:**
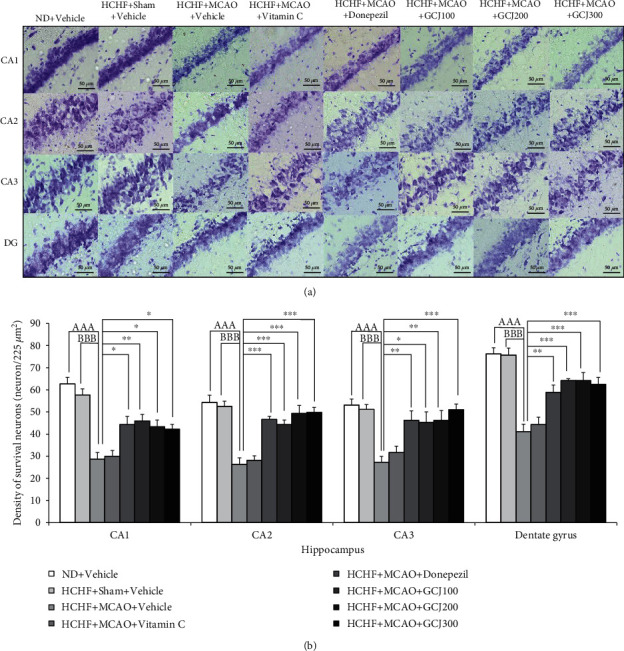
The outcome produced by the herbal formulation comprising GCJ on the survival of neuron density in the hippocampus area. (a) Representative pictures of various hippocampal regions, including CA1, CA2, CA3, and dentate gyrus, stained with cresyl violet. (b) Survival of the neuron density in CA1, CA2, CA3, and dentate gyrus of the hippocampus. Data were as stated as the mean ± SEM. ^AAA^ stands for *p* < 0.001, compared with ND+vehicle; ^BBB^ stands for *p* < 0.001, compared with HCHF diet plus sham plus vehicle; ^∗^^,^^∗∗^^,^^∗∗∗^ stand for *p* < 0.05, 0.01, and 0.001, compared with HCHF diet plus MCAO plus vehicle (*n* = 6 per each group).

Figure 4 indicates the outcome produced by GCJ on the neuronal density of surviving neurons in the prefrontal cortex. MCAO significantly reduced the neuron density in this area of HCHF plus vehicle (*p* < 0.001, compared with normal diet plus vehicle, and *p* < 0.001, compared with HCHF diet plus sham plus vehicle). Donepezil and GCJ at doses of 200 and 300 mg kg^−1^ BW attenuated the reduction of neuron density induced by MCAO in HCHF plus MCAO (*p* < 0.05, 0.01, and 0.01, respectively, compared with HCHF diet plus MCAO plus vehicle). In this study, we also evaluated the effect of GCJ on the density of the survival neurons in various regions of the hippocampus including CA1, CA2, CA3, and dentate gyrus, MetS rats subjected to MCAO.  Figure 5 shows that MCAO also reduced neuronal density in the hippocampus (CA1, CA2, CA3, and DG) of HCHF diet plus vehicle (*p* < 0.001, compared to normal diet plus vehicle, and *p* < 0.001, compared with HCHF diet plus sham plus vehicle). These changes in the areas just mentioned were mitigated by donepezil and all doses of GCJ (*p* < 0.05, 0.01, 0.05, and 0.05; *p* < 0.001 all; *p* < 0.01, 0.05, 0.01, and 0.001; *p* < 0.01, 0.001, 0.001, and 0.001, compared with HCHF diet plus MCAO plus vehicle).

### 3.4. Biochemical Changes

**Figure 6:**
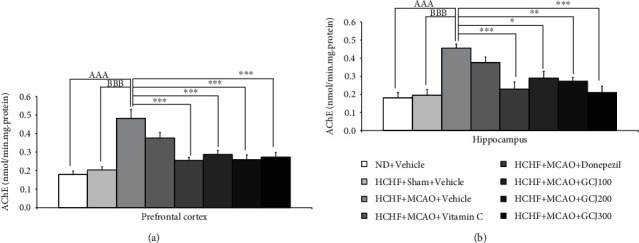
The outcome produced by the herbal formulation comprising GCJ was determined in the prefrontal area and hippocampus relative with AChE activity (a, b), respectively. Data were stated as the mean ± SEM. ^AAA^ stands for *p* < 0.001, compared with ND+vehicle; ^BBB^ stands for *p* < 0.001 compared with HCHF diet plus sham plus vehicle; ^∗^^,^^∗∗^^,^^∗∗∗^stand for *p* < 0.05, 0.01, and 0.001, compared with HCHF diet plus MCAO plus vehicle (*n* = 6 per each group).

To observe the effect of GC on the alteration of cholinergic activity, the main neurochemical system playing a role in learning and memory [25]. We also determined the effect of AChE in both the prefrontal cortex and hippocampus and the important areas in learning and memory process [26]. The outcome produced by GCJ on AChE activity in both the prefrontal cortex and hippocampus was also conducted, and data are shown in Figures 6(a) and 6(b), respectively. Our data revealed the elevation of AChE activity in both areas of HCHF diet plus MCAO plus vehicle (*p* < 0.001 all, compared with normal diet plus vehicle and *p* < 0.001, compared with HCHF diet plus sham plus vehicle). The rise of AChE activity in the prefrontal cortex of HCHF plus MCAO-treated rats was mitigated by donepezil and all doses of GCJ (*p* < 0.001, compared with HCHF diet plus MCAO plus vehicle). All treatments mentioned above also produced the same pattern of change in the hippocampus (*p* < 0.001, 0.05, 0.01, and 0.001, respectively, compared with HCHF diet plus MCAO plus vehicle).

**Figure 7:**
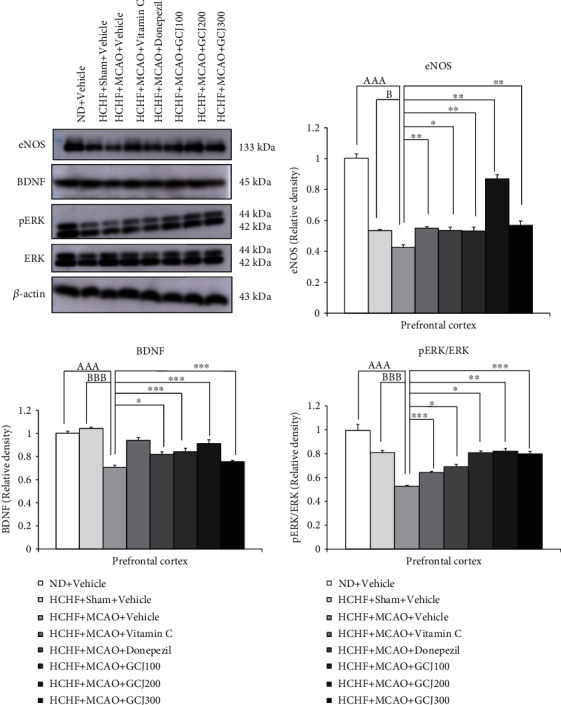
The outcome produced by the herbal formulation comprising GCJ on western blot analysis. Representative and relative densities of western blot showing the levels of eNOS, BDNF, pERK, and ERK in the prefrontal cortex, respectively. Data were stated as the mean ± SEM. ^AAA^ stands for *p* < 0.001, compared with normal diet plus vehicle; ^B,BBB^ stand for *p* < 0.05 and 0.001, compared with HCHF diet plus sham plus vehicle; ^∗^^,^^∗∗^^,^^∗∗∗^ stand for *p* < 0.05, 0.01, and 0.001 compared with HCHF diet plus MCAO plus vehicle (*n* = 6 per each group).

In Figure 7, our data revealed that the HCHF-treated rats which received sham operation and vehicle did not produce the significant changes in the expressions of BDNF, pERK/ERK, and eNOS in PFC when compared with the normal diet plus vehicle-treated rats. HCHF diet plus MCAO plus vehicle significantly reduced the expressions of eNOS, BDNF, and pERK/ERK in the prefrontal cortex (*p* < 0.001all, compared with normal diet plus vehicle, and *p* < 0.05, 0.001, and 0.001, respectively, compared with HCHF diet plus sham plus vehicle). Vitamin C produced a significant mitigation effect on the reduction of eNOS and pERK/ERK in the prefrontal cortex of HCHF diet plus MCAO (*p* < 0.01 and 0.001, compared with HCHF diet plus MCAO plus vehicle). When compared with HCHF diet plus MCAO plus vehicle, donepezil, and all doses of GCJ, they mitigated the reduction of eNOS (*p* < 0.05, 0.05, 0.001, and 0.01), BDNF (*p* < 0.05, 0.001, 0.001, and 0.001), and pERK/ERK (*p* < 0.05, 0.05, 0.01, and 0.001).

**Figure 8:**
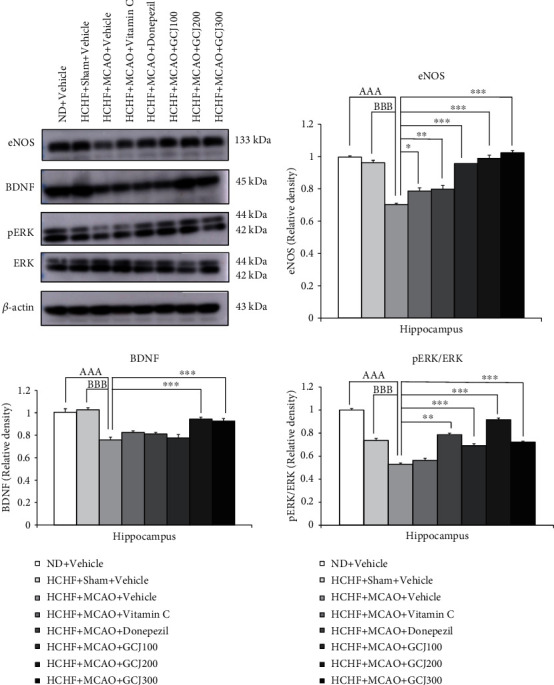
The outcome produced by the herbal formulation comprising GCJ on western blot analysis. Representative and relative densities of western blot exhibiting the levels of eNOS, BDNF, pERK, and ERK in hippocampus, respectively. Data were stated as the mean ± SEM. ^AAA^ stand for *p* < 0.001 compared with normal diet plus vehicle; ^B,BBB^ stand for *p* < 0.05 and 0.001 compared with HCHF diet plus sham plus vehicle; ^∗^^,^^∗∗^^,^^∗∗∗^ stand for *p* < 0.05, 0.01, and 0.001, compared with HCHF diet plus MCAO plus vehicle (*n* = 6 per each group).

The effect of GCJ on the expressions of all parameters mentioned earlier in the hippocampus was also determined, and results are shown in Figure 8. The outcome produced by GCJ on the aforementioned parameters in hippocampus are also explored, and the cause produced is shown in Figure 8. The MCAO procedure resulted in significant reduction in the expression of eNOS, BDNF, and pERK/ERK in the hippocampus of HCHF diet plus vehicle (*p* < 0.001 all, compared with normal diet plus vehicle, and *p* < 0.001 all, compared with HCHF diet plus sham plus vehicle). Vitamin C significantly mitigated the reduction of eNOS expression, whereas donepezil and a low dose of GCJ significantly mitigated the reduction of eNOS and pERK/ERK expressions in the hippocampus of HCHF diet plus MCAO (*p* < 0.01 all and 0.001 all, respectively, compared with HCHF diet plus sham plus vehicle). Conversely, the medium and high administered doses of GCJ significantly mitigated the reduction of eNOS, BDNF, and pERK/ERK expressions in the hippocampus of HCHF diet plus MCAO (*p* < 0.001 all, compared with HCHF diet plus MCAO plus vehicle).

## 4. Discussion

The present research findings clearly illustrated that “GCJ”, a functional ingredient, improves spatial and nonspatial memory in cerebral ischemic rats with metabolic syndrome. It also improves the survival of neurons, especially in the areas that contribute an essential role in learning and memory, such as the hippocampus. The decrease in the activity of AChE and the increase in the expressions of eNOS, BDNF, and pERK/ERK in both areas mentioned earlier are also observed.

Our data reveal that vitamin C, a well-known antioxidant used in this study, fails to show the positive modulation effect on all parameters monitored in this study. The discrepancy in these data from our previous work may be associated with the poor shelf life of the drug after long-term storage. In addition, the variation in temperature of the storage condition, donepezil, a standard drug, still produces positive modulation effects on all parameters used in this study.

It has been revealed that the hippocampus is accountable for learning and memory, whereas the prefrontal cortex (PFC) plays a crucial role in working memory. In addition, the hippocampal-prefrontal cortex (Hip-PFC) circuit also plays a substantial function in cognitive and memory consolidation [27]. A recent report demonstrates that prefrontal–hippocampal networks with cholinergic exerts a pivotal role on brain synaptic plasticity and memory process [9]. Interestingly, our data clearly demonstrate that GCJ increases neuron density in both the prefrontal cortex and hippocampus but suppresses AChE activity in the areas mentioned. In addition, GCJ also increases both spatial and nonspatial memory performance. Therefore, these data suggest that GCJ increases the functional circuit of Hip-PFC and the interaction of prefrontal–hippocampal networks with cholinergic which in turn increase brain synaptic plasticity and memory process, particularly encoding, consolidation, and retrieval process [27–29]. Our findings also confirm the previous findings [30–32].

However, brain plasticity and memory processes are under the regulation of many factors including brain-derived neurotrophic factor [7, 10] and cerebral blood flow [33–35]. A reduction in cerebral blood flow can induce deterioration in memory performance [34], whereas an increase in cerebral blood flow can improves memory performance [35]. It has been reported that cerebral blood flow is tightly associated with endothelial nitric oxide synthase (eNOS), a key enzyme for synthesizing a main vasorelaxation agent such as nitric oxide [36, 37]. The activation of the nitric oxide signaling process is also under the influence of acetylcholine, a physiological stimulator [37]. In this study, we have demonstrated that GCJ suppresses AChE which in turn increases ACh and increases the expression of eNOS in the prefrontal cortex and the hippocampus. Therefore, GCJ can enhance memory performance partly via the suppression of AChE, giving rise to the elevation of ACh, which stimulates the expression of eNOS in both areas mentioned earlier, resulting in the increase in memory performance. However, the regulation of eNOS is also under the influence of many factors. Our data show that vitamin C increase eNOS expression in both areas but failed to suppress AchE. Therefore, an increase in eNOS expression in the mentioned areas of HCHF-treated rats that were subjected to MCAO and vitamin C may occur due to fluid shear stress, VEGF, and bradykinin [38]. Unfortunately, these factors are not monitored in this study, and this requires further investigation.

BDNF plays a crucial role not only in the memory process but also in the survival of neurons. Its action is associated with activating the extracellular signal-regulated kinase (ERK) signaling pathway [39, 40]. The activation of this pathway leads to an increase in its kinase activity, giving rise to the enhancement of the transcription process of many proteins, including proteins essential for the proliferation and survival of neurons [41]. These data corresponds with the current data which demonstrates the reduction in both BDNF and the expression of pERK/ERK together with the decrease in survival neurons in the prefrontal cortex and all investigated hippocampus regions in cerebral ischemia rat with MetS. The current data reveal that GCJ enhances the expressions of BDNF and pERK/ERK in the prefrontal cortex and the hippocampus. These changes correspond with the increase in neuron density and memory performance. Therefore, the memory enhancer effect of GCJ may also occur as the results of an elevation of BDNF which intern increases in pERK/ERK expression, and increases in neuron density and memory performance. Furthermore, the elevation in BDNF and pERK/ERK signaling molecule can also increase eNOS expression [42]. Thus, GCJ can also enhance BDNF, leading to an elevation of eNOS expression.

Taken all together, the current study demonstrates an increase in BDNF, which in turn increases brain plasticity, and eNOS, which in turn increases in neuron density in the prefrontal cortex and hippocampus. GCJ also suppresses AChE, giving a rise to an increase in ACh in both areas just mentioned. The increase in both neuron density and cholinergic function gives rise to an elevation in the Hip-PFC functional circuit which plays an important role in learning and memory. Moreover, an elevation in cholinergic function can also increase memory performance. Currently, the epigenetic mechanisms are controlling activity-dependent gene transcription leading to regulate synaptic plasticity in the brain, eNOS and memory function [43, 44]. In addition, signal transduction via ERK influences on epigenetic mechanisms [45]. Therefore, it may be possible that GCJ modulates signal transduction of ERK by increasing pERK/ERK which in turn modulates epigenetic mechanism in brain plasticity encouraged by BDNF, eNOS, and an improvement of cholinergic function in the prefrontal cortex and hippocampus. Unfortunately, we did not measure the alteration of epigenetic mechanisms in this study. However, it is worth further exploring the neurogenomic effect of GCJ to establish the clear connection between epigenetic mechanisms and all the changes observed in this study.

## 5. Conclusions

The functional ingredient of GCJ can increase the expressions of eNOS, BDNF, and pERK/ERK and enhance neuron density and neural plasticity in the prefrontal cortex and hippocampus. All of the factors mentioned earlier can improve memory performance, antidementia effect following ischemic stroke with the metabolic syndrome, and neuroprotective against stroke. However, this required confirmation by a clinical study.

## Figures and Tables

**Table 1:** Group division of the experimental animals in the study.

Group	Name	Description
I	ND+vehicle	Rats were served food with normal diet and administered with vehicle.
II	HCHF+sham+vehicle	Rats were induced MetS, received sham surgery, and administered with vehicle.
III	HCHF+MCAO+vehicle	Rats were induced MetS, received MCAO surgically, and administered with vehicle.
IV	HCHF+MCAO+vitamin C	Rats were induced MetS, received MCAO surgically, and administered with vitamin C at the dose of 250 mg kg^−1^ BW.
V	HCHF+MCAO+donepezil	Rats were induced MetS, received MCAO surgically, and administered with donepezil at the dose of 3 mg kg^−1^ BW.
VI	HCHF+MCAO+GCJ100	Rats were induced MetS, received MCAO surgically, and administered with GCJ at the dose of 100 mg kg^−1^ BW.
VII	HCHF+MCAO+GCJ200	Rats were induced MetS, received MCAO surgically, and administered with GCJ at the dose of 200 mg kg^−1^ BW.
VIII	HCHF+MCAO+GCJ300	Rats were induced MetS, received MCAO surgically, and administered with GCJ at the dose of 300 mg kg^−1^ BW.

HCHF: high-carbohydrate high-fat diet; MCAO; reperfusion injury (I/R) at the right middle cerebral artery.

**Table 2:** The main ingredients presented in GCJ.

GCJ	HPLC phytochemical
6-Gingerol	36.981 ± 0.62
6-Shogaol	8.558 ± 0.19
Catechin	0.137 ± 0.04
Chlorogenic acid	0.042 ± 0.007
Caffeic acid	0.029 ± 0.001

## Data Availability

The data used to support the findings of this study are available from the corresponding author upon request.

## Abbreviations

AChE: Acetylcholinesterase
BDNF: Brain-derived neurotrophic factor
eNOS: Endothelial nitric oxide synthase
GCJ: Polyherbal functional ingredient containing ginger, Chinese date, and wood ear mushroom
HCHF: High carbohydrate high fat diet
MCAO: Middle cerebral artery occlusion
MetS: Metabolic syndrome
NOR: Novel object recognition
pERK/ERK: Phosphorylated extracellular signal-regulated kinase/extracellular signal-regulated kinase.

## Supplementary Materials

Supplementary Materials Supplementary materials about the dataset of the original western blot carried out in this study are attached as supplementary material.
