# Supplementary material for: The Polyherbal Functional Ingredient Containing Ginger, Chinese Date, and Wood Ear Mushroom Protects against Dementia following Metabolic Syndrome
Source: Biomed Res Int. 2023 Aug 2;2023:9911397. doi: 10.1155/2023/9911397 (PMC10412205; doi:10.1155/2023/9911397)
Supplement: Supplementary Materials — about the dataset of the original western blot carried out in this study are attached as supplementary material. [file 9911397.f1.pdf]

Supplementary Data (Prefrontal Cortex)

Antibodies	Repeat 1	Repeat 2	Repeat 3
eNOS (133 kDa)	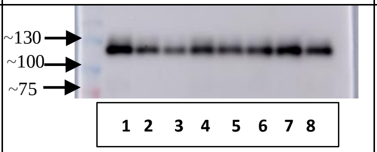	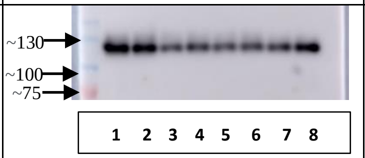	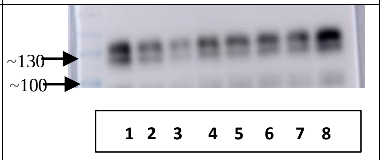
BDNF (45 kDa)	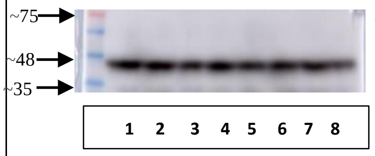	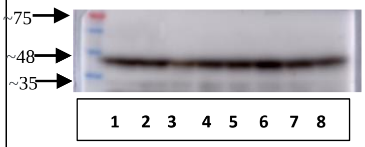	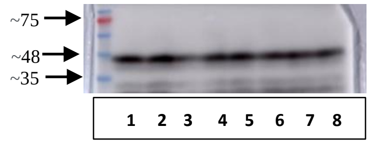
pERK (44 kDa) (42 kDa)	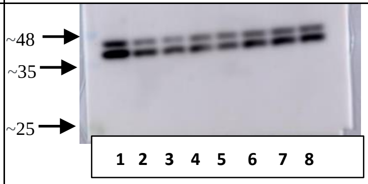	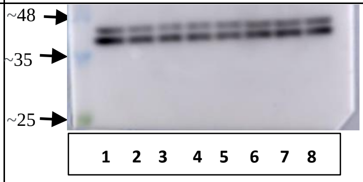	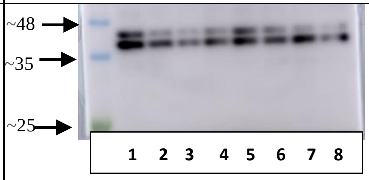
ERK (44 kDa) (42 kDa)	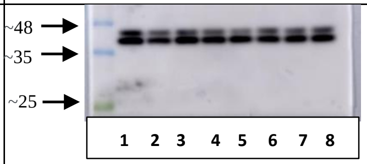	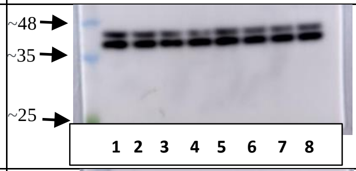	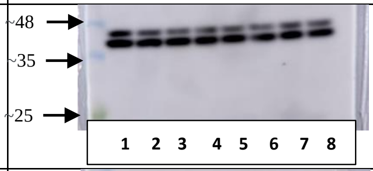
β-actin (43 kDa)	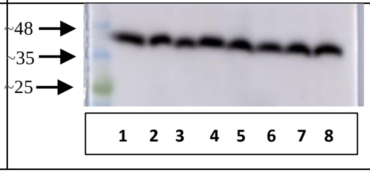	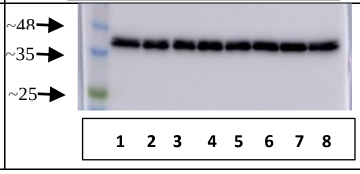	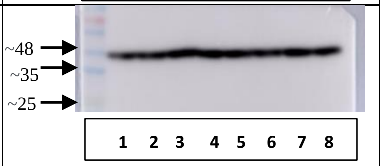

Lane1; ND+Vehicle
Lane2; HCHF+Sham+Vehicle
Lane3; HCHF+MCAO+Vehicle
Lane4; HCHF+MCAO+VitaminC
Lane5; HCHF+MCAO+Donepezil
Lane6; HCHF+MCAO+GCJ100
Lane7; HCHF+MCAO+GCJ200
Lane8; HCHF+MCAO+GCJ300

Supplementary Data (Hippocampus)

**Antibodies**	**Repeat 1**	**Repeat 2**	**Repeat 3**
eNOS (133 kDa)	~130 ~100 ~75 ~63 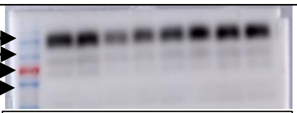 1 2 3 4 5 6 7 8	~130 ~100 ~75 ~63 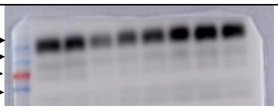 1 2 3 4 5 6 7 8	~130 ~100 ~75 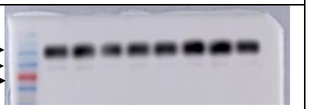 1 2 3 4 5 6 7 8
BDNF (45 kDa)	~75 ~63 ~48 ~35 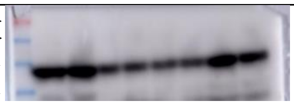 1 2 3 4 5 6 7 8	~75 ~48 ~35 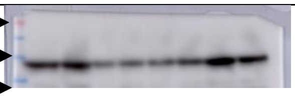 1 2 3 4 5 6 7 8	~75 ~48 ~35 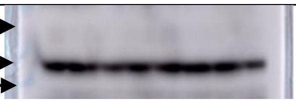 1 2 3 4 5 6 7 8
pERK (44 kDa) (42 kDa)	~48 ~35 ~25 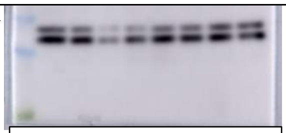 1 2 3 4 5 6 7 8	~48 ~35 ~25 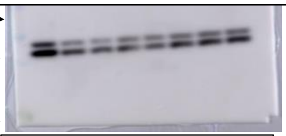 1 2 3 4 5 6 7 8	~48 ~35 ~25 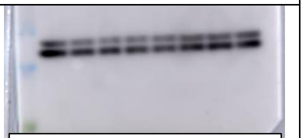 1 2 3 4 5 6 7 8
ERK (44 kDa) (42 kDa)	~75 ~63 ~48 ~35 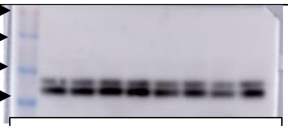 1 2 3 4 5 6 7 8	~75 ~63 ~48 ~35 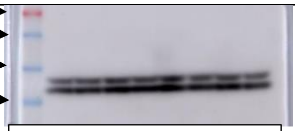 1 2 3 4 5 6 7 8	~75 ~63 ~48 ~35 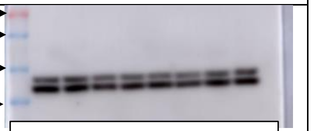 1 2 3 4 5 6 7 8
β-actin (43 kDa)	~63 ~48 ~35 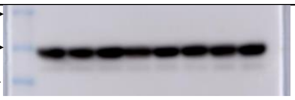 1 2 3 4 5 6 7 8	~63 ~48 ~35 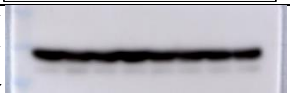 1 2 3 4 5 6 7 8	~63 ~48 ~35 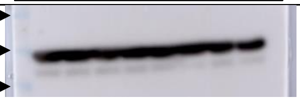 1 2 3 4 5 6 7 8

Lane1; ND+Vehicle
Lane2; HCHF+Sham+Vehicle
Lane3; HCHF+MCAO+Vehicle
Lane4; HCHF+MCAO+VitaminC
Lane5; HCHF+MCAO+Donepezil
Lane6; HCHF+MCAO+GCJ100
Lane7; HCHF+MCAO+GCJ200
Lane8; HCHF+MCAO+GCJ300
